# One-stage cartilage repair using the autologous matrix-induced chondrogenesis combined with simultaneous use of autologous adipose tissue graft and adipose tissue mesenchymal cells technique: clinical results and magnetic resonance imaging evaluation at five-year follow-up

**DOI:** 10.1007/s00264-023-05921-8

**Published:** 2023-09-01

**Authors:** Fabio Valerio Sciarretta, Claudio Ascani, Luca Sodano, Carolina Fossati, Silvana Campisi

**Affiliations:** 1 Clinica Nostra Signora della Mercede, Via Tagliamento 25, 00198 Rome, Italy; 2 Accademia Biomedica Rigenerativa (ABRI), Via Misurina 56, 00135 Rome, Italy; 3 Artemisia Lab, Via Piave 76, 00198 Rome, Italy; 4 Ospedale CTO, Via San Nemesio 28, 00145 Rome, Italy; 5 Ospedale San Luca, Via Francesco Cammarota, 84078 Vallo della Lucania, SA Italy

**Keywords:** LIPO-AMIC, Cartilage defects, Cartilage repair, Adipose tissue graft, Adipose tissue stem cells, Matrix-induced chondrogenesis

## Abstract

**Purpose:**

To evaluate medium-term outcomes of knee cartilage defects repair by autologous matrix-induced chondrogenesis combined with simultaneous use of autologous adipose tissue graft and adipose tissue mesenchymal cells, defined as LIPO-AMIC technique.

**Methods:**

The LIPO-AMIC technique has been used in ICRS degree III–IV knee defects. Eighteen patients have been prospectively evaluated during two and five years both clinically and by MRI.

**Results:**

Patients showed progressive significant improvement of all scores starting early at six months, and further increased values were noted till the last follow-up at 60 months. Mean subjective pre-operative IKDC score of 36.1 significantly increased to 86.4 at 24 months and to 87.2 at 60 months. Mean pre-operative Lysholm score of 44.4 reached 93.5 at two years and 93.5 at five years. MRI examination showed early subchondral lamina regrowth and progressive maturation of repair tissue and filling of defects. The mean total MOCART score showed that a significative improvement from two year follow-up (69.1 points) to last follow-up was 81.9 points (range, 30–100 points, SD 24). Complete filling of the defect at the level of the surrounding cartilage was found in 77.8%.

**Conclusions:**

Adipose tissue can represent ideal source of MSCs since easiness of withdrawal and definite chondrogenic capacity. This study clearly demonstrated the LIPO-AMIC technique to be feasible for treatment of knee cartilage defects and to result in statistically significant progressive clinical, functional and pain improvement in all treated patients better than what reported for the AMIC standard technique, starting very early from the 6-month follow-up and maintaining the good clinical results more durably with stable results at mid-term follow-up.

## Introduction

Focal articular cartilage defects are very common in the knee joint, causing important joint disfunction associated to pain, swelling, limited motion and walking. In 2007, Widuchowski et al. [1], in their retrospective study on 25,124 patients, found that the prevalence of knee chondral lesions was 60% in patients undergoing knee arthroscopies. Of these, 67% were classified as focal chondral or osteochondral lesions following Outerbridge classification, mostly localised in the patella (36%) and in the medial femoral condyle (34%). This prevalence report from a very large database confirmed the 63% previously reported incidence of chondral lesions encountered in patients undergoing knee arthroscopies [2].

Although cartilage injuries are frequent findings, cartilage regenerative treatments guaranteeing long-term durable results are still very limited. Various treatments have been proposed in order to impede lesions degeneration and predisposition to OA. In fact, to prevent further degeneration and to improve clinical symptoms, cell-based treatments as autologous chondrocyte transplantation (ACI) and matrix-induced autologous chondrocyte implantation (MACI) require a two step procedure to first harvest chondrocytes for incubation in the laboratory followed by implantation in a second step surgery. Conversely, available one step procedures include osteochondral autografts (OATS and mosaicplasty), osteochondral allograft implantation (OCA), bone marrow stimulation techniques and autologous matrix-induced chondrogenesis (AMIC).

Among the reparative procedures proposed, the Autologous Matrix-Induced Chondrogenesis (AMIC®) one-step technique introduced by Benthien and Behrens [3], combining microfracturing with a collagen I/III Matrix (Chondro-Gide®, Geistlich Pharma AG, Wolhusen, Switzerland), demonstrated its clinical and cost-effectiveness and safety with no donor site morbidity also in the long-term follow-up meta-analysis, superior to simple bone marrow stimulation [4, 5].

Although effective for many patients, recent research focused on improving clinical outcome scores, preventing OA progression and reducing recovery times through the use of biologics. This has brought to validate one-step procedures based on bone marrow aspirate concentrate or adipose tissue graft containing adipose tissue MSCs [6] pasted on the cartilage defect and covered with a commercially available collagen I/III scaffold to augment the results by providing a stronger chondrogenic environment and exploiting MSC paracrine action.

In 2017, the authors already published a pilot study which described the development, the technical steps and the preliminary outcomes of autologous matrix-induced chondrogenesis (AMIC) combined with the simultaneous use of autologous adipose tissue graft–derived mesenchymal stem cells, defined as LIPO-AMIC technique, for the treatment of knee full thickness cartilage defects, following exactly the same indications originally proposed for the standard AMIC technique for the treatment of grade III or IV cartilage lesions according to the ICRS (International Cartilage Repair Society) classification, ranged between 2 and 8 cm^2^.

The purpose of this study was to update our experience and evaluate the medium-term clinical results of cartilage regeneration using the one-step technique of LIPO-AMIC for the treatment of full thickness cartilage defects in the knee [7].

## Methods

In this study, the initial 18 physically active consecutive patients with one symptomatic focal knee chondral defect major than 10 mm diameter treated with the LIPO-AMIC technique and object of the 2017 pilot study by same authors were followed prospectively until they all reached five years of follow-up. All the patients included in this study were informed in detail of the surgical technique and provided their authorization for the intervention by signing the informed consent.

No patient was lost at final follow-up. Preliminary data of this patient’s cohort have been reported previously [7].

Candidates for operation met the following inclusion criteria: age between 30 and 60 years, symptomatic grade III–IV cartilage lesions according to the classification of the International Cartilage Regeneration and Joint Preservation Society (ICRS), defects to be treated of grade III and IV greater than 1 cm^2^ in size and availability for two and five year follow-up assessments.

The main exclusion criteria were advanced knee osteoarthritis, significant narrowing of the femoro-tibial joint spacing, pre-existing rheumatic disease, previous total meniscectomy, significant overweight (BMI > 35), presence of malalignment or tibio-femoral or patello-femoral joint instability which cannot be treated concomitantly.

The study protocol was approved by the local institutional ethics committee, and informed consent was obtained from all patients taking part in the study.

### Clinical and radiographic evaluation

Cartilage lesions have always been for each patient highlighted with preoperative magnetic resonance imaging (MRI) and confirmed by knee arthroscopy which has also allowed the diagnosis as grade III or IV injuries according to the ICRS classification and the definition of the final size and shape of the defect to be treated. The lesions were located in the medial or lateral femoral condyle, the retrosurface of the patella or the femoral trochlea. Coexisting pathologies were treated concurrently during the same surgical procedure.

All the patients followed the same post-operative rehabilitation protocol, differentiated according to the site of the lesion, and were evaluated preoperatively and followed up with regular clinical checks, every 15 days for the first four months, then at six months, 12 months and at two and five years and using magnetic resonance examinations of the knee. All patients were regularly assessed, pre-operative and post-operative, in a non-blinded manner by the treating surgeon according to the International Knee Documentation Committee (IKDC) Knee Examination Form, with a final functional grade of normal, nearly normal, abnormal or severely abnormal given based on the findings of effusion, passive motion deficit and ligamentous stability. Patient-reported scoring tools consisted of the IKDC Subjective Knee Evaluation, the Lysholm Knee Score, the Knee Injury and Osteoarthritis Outcome Score (KOOS) and the Visual Analogue Scales (VAS) for pain assessment (use of a 10-cm graded line, with 0 indicating no pain and 10 indicating the worst pain imaginable), up to 60 months of follow-up.

Radiographic examinations were performed for each patient with standard antero-posterior and lateral weight-bearing knee radiographs and knee magnetic resonance imaging (MRI). Preoperative MRI was performed to determine size, location and classification of the lesions. Postoperative MRI is considered the most important non-invasive method for evaluation of surgical procedures for cartilage repair. MRI examinations were therefore undertaken at follow-ups. For the description of the repair tissue, we used the MOCART (Magnetic Resonance Observation of Cartilage Repair Tissue) system, introduced in 2004 by Marlovits et al. [8] and based on a standard knee MRI protocol including intermediate-weighted sequences for cartilage evaluation. Originally, the score was designed for the evaluation of cartilage repair tissue after microfracturing, ACI or MACI in the knee.

MOCART score is an objective evaluation based on nine radiologic parameters (degree of repair and defect filling, integration to border tissue, surface of repair tissue, structure of repair tissue, subchondral lamina, subchondral bone, adhesions, synovitis) describing the morphology and signal intensity of the repair tissue, compared with the native cartilage. The repair evaluation includes the thickness of the tissue, integration of the margins, smoothness of joint surfaces and the subchondral bone status [8]. All MRI images and pre-operative and postoperative clinical function were evaluated by an independent reviewer.

### Operative technique

All surgical procedures were performed in a single surgical step, under loco-regional anaesthesia and after routine preparation of the sterile operating field.

**Fig. 1:**
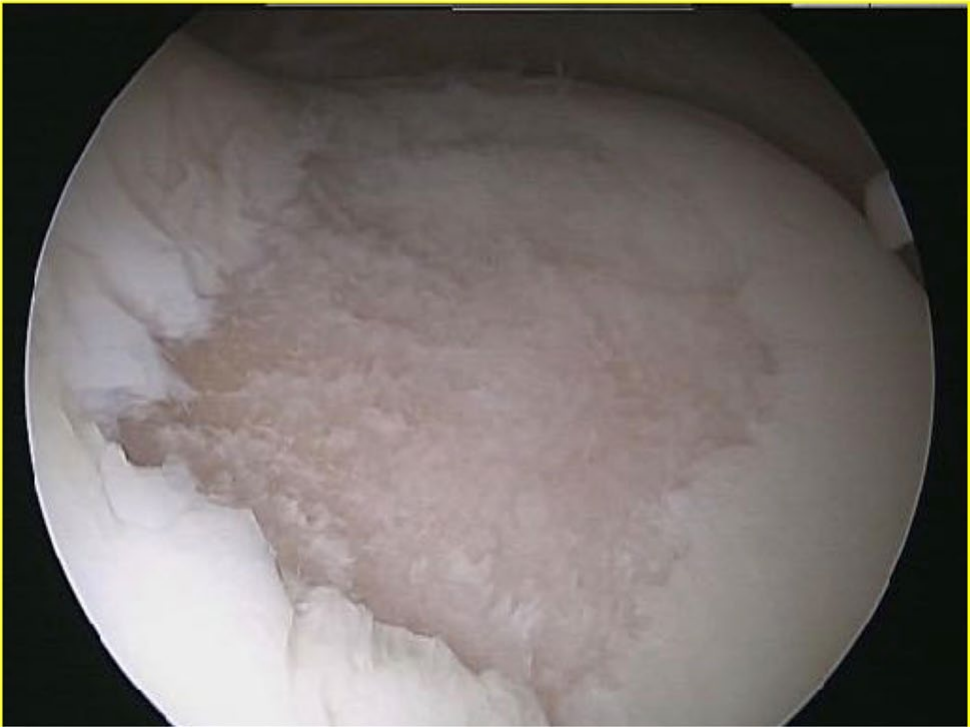
Full-thickness cartilage defect of the trochlea

Arthroscopic evaluation was performed to visualise the cartilage defects and to confirm magnetic resonance imaging findings with respect to location and size of the lesions (Fig. 1).

Subsequently, under local anaesthesia of the abdomen, after infiltration of Klein’s solution, 50–80 ml of lipoaspirate were extracted by the simple method of liposuction from the periumbilical adipose tissue, using a dedicated disposable kit available on the market for the suction and subsequent processing (filtration and micro-fragmentation) and grafting of adipose tissue (Process Kit Lipogems, Lipogems International SpA, Milan, Italy), for the use of which we have followed the instructions provided by the manufacturer. This device progressively reduces the size of the adipose tissue clusters, at the same time eliminating blood residues and oily substances with pro-inflammatory capacities, minimizing, thanks to the carrying out of the whole process inside physiological solution, the risks of damaging the mesenchymal cells.

Once the adipose harvesting and the subsequent processing step were completed, having obtained 10 ml adipose graft containing adipose tissue mesenchymal cells (ADSCs) and SVF cells ready to use in the syringe contained in the processing kit, the following step of the surgery was represented by the repair of the focal chondral or osteochondral defect. This operative procedure was performed through a mini-open approach as described previously [7].

Once identified, the chondral or osteochondral defect was carefully debrided with curettes and chondrectomes until stable, clean and perpendicular edges of healthy cartilage were obtained and then accurately measured.

**Fig. 2:**
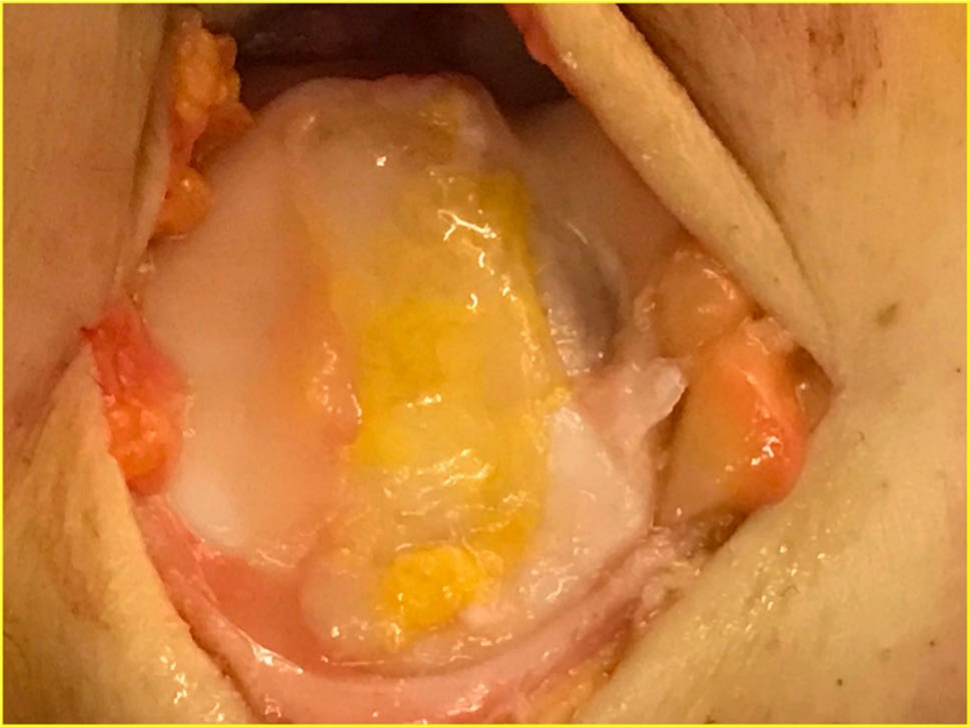
Final view of full-thickness defect of the trochlea treated by the LIPO-AMIC technique

**Fig. 3:**
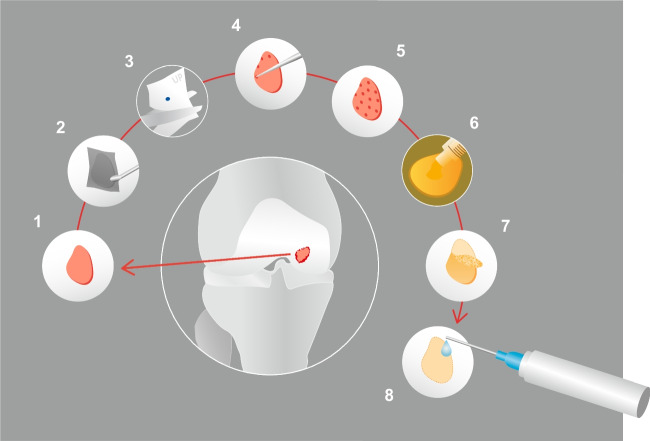
LIPO-AMIC technique. Following knee arthroscopy and adipose tissue lipoaspiration, the LIPO-AMIC procedure is performed with either an arthrotomy or arthroscopy. (1) The defect is carefully debrided with curettes and chondrectomes. (2) A perfect copy of the defect is obtained by aluminium template. (3) The membrane is cut in the right size on the basis of the template. (4, 5) Microfractures are performed from the periphery towards the centre of the defect. (6) The adipose tissue graft and ADSCs are applied onto the defect. (7) The type I/III enriched collagen membrane, already soaked for 10–15 min in adipose tissue graft, is implanted with its porous layer facing the subchondral bone and fixed with fibrin glue, followed by further application of ADSCs and adipose tissue graft on top of the repair

A perfect copy of the defect was then obtained using an aluminium model, from which the exact imprint of the defect was cut out. Microfractures were carefully performed, using a chondro pick in the subchondral bed of the lesion until bleeding is observed, taking care to distribute them evenly from the periphery to the centre and always respecting the distance between one hole and the other in order to leave healthy and resistant bone bridges. Subsequently, the membrane (Chondro-Gide®, Geistlich Pharma AG, Wolhusen, Switzerland) was cut on the basis of the aluminium cut model and immersed for a few minutes in 4–5 ml of the micro-fragmented adipose tissue and in the stromal vascular fraction graft extracted from the adipose tissue. The collagen membrane constitutes an ideal scaffold as its porous nature allows the cells to nest inside the scaffold, thus finding the most suitable environment for growth and differentiation. At this point, the enriched membrane was inserted into the joint to directly and accurately cover the defect with the smooth side facing up. The remaining part of the cells and adipose tissue graft obtained from the adipose tissue (5–6 ml) was infiltrated on the site of the lesion, and then, membrane and adipose graft and MSCs were sealed and secured to surrounding cartilage by use of surgical fibrin glue (Tissucol, Baxter SpA, Rome, Italy) (Figs. 2 and 3). In this way, the membrane will perform a double action: In addition to representing the barrier capable of retaining the mesenchymal cells from the medullary blood, it constitutes a scaffold enriched and activated by the mesenchymal adipose cells to accelerate the process of chondrocyte development.

After positioning the Chondro-Gide® bilayer collagen membrane and waiting a few minutes to be sure of its adhesion, knee flexion-extension movements were repeatedly performed from complete flexion to complete extension to check the stability of the implant and ensure proper graft fixation and the surgical wounds were closed as routine, without applying any drain, and the knee immobilised in a soft bandage.

The coexisting pathologies if present (tibio-femoral malalignments, patello-femoral malalignments and ligament laxities) have been always treated concurrently during the same one-step surgical procedure.

The knee joint was finally closed with Vicryl suturing, and the wound was closed in layers with resorbable sutures.

### Rehabilitation protocol

All patients have followed the same rehabilitation protocol under the supervision of a physical therapist. The early period was devoted to pain control, reduce possible effusion, gain and maintain range of motion and quadriceps muscle strengthening. Range of motion and weightbearing protocols were differentiated depending on defect location, on the femoral condyle or the patello-femoral joint. In patello-femoral joint, progressive weightbearing with crutches was immediately allowed, limiting ROM from 0 to 60° of flexion for the first three weeks. In femoral condyle defects, weight bearing was restricted for the initial four weeks postoperatively, achieving unrestricted complete weightbearing generally by six weeks. Great importance was emphasised on early regaining of normal gait patterns.

### Statistical analysis

We used the two-tailed *t*-test equal variance to analyse statistical differences between preoperative and six to 12–24–60-month follow-up KOOS, IKDC, LYSHOLM, VAS scores and pre–operative to 24–60-month MOCART score. A *p*-value <0.05 was considered statistically significant. Data were analysed with SPSS software version 17.0.

## Results

Eighteen patients with full thickness chondral defects of the knee were treated with the LIPO-AMIC procedure. The average age was of 40.2 years (range, 31 to 58 years). The sex ratio between males and females was 2.6:1 (13 males, 5 females). The disease was localised on the right knee in eight cases and on the left knee in ten.

Twelve patients had suffered a previous sport-related injury, three patients had experienced an injury while working and three patients could not recall any significant trauma prior to the onset of symptoms. No patient had undergone previous surgeries. In terms of the individual zones, six (33.3%) lesions were located in the medial femoral condyle, six (33.3%) lesions were located in the patella, four lesions (22.2%) were located in the femoral trochlea and two (11.1%) lesions were located in the lateral femoral condyle. Lesions were classified according to ICRS classification: six (33.3%) lesions were classified as stage III, where a loss of more than 50% of chondral substance depth is present and, in some areas, the subchondral bone surface is reached but not passed through; 12 (66.6%) lesions were classified as stage IV, extensive full thickness chondral defects. When performing the LIPO-AMIC procedure, the coexisting pathologies were treated concurrently during the same surgical procedure. Specifically, partial meniscectomies (*n* = 2), patella realignment surgery (*n* = 2) and anterior cruciate ligament reconstruction ( *n*= 1) were performed (Table 1).

**Table 1:** Epidemiological results

Epidemiological results	Patients (*n*)
*Location*	
*(Medial femoral condyle)*	6
*(Lateral femoral condyle)*	2
*(Patella)*	6
*(Femoral trochlea)*	4
*Related injury/no injury*	15/3
*ICRS preoperative*	
*Stage III*	6
*Stage IV*	12
*Coexisting pathologies*	
*Partial meniscectomies*	2
*Patella realignment surgery*	2
*ACL reconstruction*	1
*Intraoperative complications*	0
*Wound infection*	0

We had no intraoperative complications. No early or late postoperative complications were observed in any of the patients. There were no cases of superficial wound infection. No significant osteoarthritic changes were showed at the last follow-up MRI. No patient treated by index procedure has required additional surgeries or has underwent in 5-year follow-up period to knee arthroplasty.

### Functional outcome

All 18 patients with chondral injuries treated with the LIPO-AMIC technique included in the final analysis were followed prospectively and were evaluated both clinically and by magnetic resonance imaging with progressive follow-ups at six and 12 months and were all available for follow-up assessments at two and five years postoperatively.

Patients were assessed by IKDC, Lysholm, KOOS and VAS scores.

The average age of patients, five females and 13 males, was 40.2 ± 2.8 years (range 31–58).

The mean defect size of the chondral lesions was 3.1 ± 1.1 cm^2^ (range 1, 3–4, 5)

The BMI of the patients was equal to 25.5 ± 2.1 (range 18–33) (Table 2).

**Table 2:** Patients baseline characteristics and demographics

Baseline data	Patients (*n* 18)
Males/females	13/5
Left/right	10/8
Follow-up *(months)*	60
Age at surgery *(mean years) (range)*	40.2 (31–58)
BMI *(mean value) (range)*	25.5 (18–33)
Cartilage lesion *(mean size cm*^*2*^*) (range)*	3.1 (1.3–4.5)

All patients showed significant improvements in all scores at six and 12-month follow-ups and at two and five year follow-up controls (Table 3).

**Table 3:** Statistical evaluation of clinical outcome scores (IKDC, LYSHOLM, KOOS and VAS) pre-op and at 6 months, 12 months, 2-year and 5-year follow-up

	Follow-up					*P* value	
	Preoperative	6 months	12 months	24 months	60 months	Preoperative/6–12–24–60 months	24/60 months
VAS						*p* < 0.05	*p* = 0.52
*Mean value, SD*	6.5 ± 0.7	1.1 ± 0.7	0.8 ± 0.7	0.7 ± 0.7	0.8 ± 08		
*(Range)*	(5–8)	(0–3)	(0–3)	(0–3)	(0–2)		
IKDC						*p* < 0.05	*p* = 0.75
*Mean value, SD*	36.1 ± 7.1	78.7 ± 6.4	82 ± 4.9	86.4 ± 6.7	87.2 ± 7		
*(Range)*	(24–44)	(60–90)	(66–92)	(68–94)	(67–94)		
LYSHOLM						*p* < 0.05	*p* = 0.98
*Mean value, SD*	44.4 ± 10.5	83.2 ± 3.5	87.7 ± 2.8	93.5 ± 0.7	93.5 ± 0.7		
*(Range)*	(26–66)	(65–93)	(66–96)	(69–98)	(68–98)		
KOOS pain						*p* < 0.05	*p* = 0.84
*Mean value, SD*	64.1 ± 9.2	88.9 ± 8.4	93.1 ± 4.9	94.2 ± 3.5	93.7 ± 3.5		
*(Range)*	(51–72)	(73–100)	(80–100)	(80–100)	(80–100)		
KOOS sympt						*p* < 0.05	*p* = 0.78
*Mean value, SD*	66 ± 4.7	86.8 ± 5.6	90.9 ± 3.6	92.5 ± 5	92.1 ± 1.4		
*(Range)*	(54–72)	(68–95)	(82–95)	(82–100)	(85–100)		
KOOS adl						*p* < 0.05	*p* = 0.81
*Mean value, SD*	68.1 ± 1.4	90.9 ± 2.8	94.7 ± 2.8	95.1 ± 2.8	94.8 ± 4.2		
*(Range)*	(56–77)	(78–100)	(85–100)	(85–100)	(88–100)		
KOOS sport						*p* < 0.05	*p* = 0.80
*Mean value, SD*	43.6 ± 7	68.5 ± 4.9	72.9 ± 8.5	74.4 ± 8.8	75.2 ± 7.9		
*(Range)*	(28–55)	(45–85)	(60–93)	(60–90)	(65–93)		
KOOS qol						*p* < 0.05	*p* = 0.94
*Mean value, SD*	38.7 ± 3.5	76.3 ± 12	77–9 ± 14.1	82.4 ± 3.5	82.1 ± 3.5		
*(Range)*	(30–53)	(63–100)	(64–95)	(60–100)	(65–100)		

No adverse reactions or intra and postoperative complications were observed.

The mean pre-operative value of the subjective IKDC score was equal to 36.1 ± 7.1 (range 24–44) and significantly increased to a mean value at six months equal to 78.7 ± 6.4 (range 60–90), at 12 months to 82 ± 4.9 (range 66–92 ) and 24 months at 86.4 ± 6.7 (range 68–94) and 87.2 ± 7 (range 67–94) at 60 months.

The mean pre-operative value of the Lysholm Knee Score of 44.4 ± 10.5 (range 26–66) reached a value of 83.2 ± 3.5 (range 65–93) at six months and of 87.7 ± 2.8 (range 66–96) at the 12-month follow-up and of 93.5 ± 0.7 (range 69–98) at two year follow-up and the value of 93.5 ± 0.7 (range 68–98) at final follow-up at five years.

The KOOS score broken down into subgroups showed the following results.

The pain assessment score went from a pre-operative score of 64 ± 9.2 (range 51–72) to 88.9 ± 8.4 (range 73–100) at six months, 93 ± 4.9 (range 80–100) at 12 months, 94.2 ± 3.5 (range 80–100) at 24 months and 93.7 ± 3.5 (range 80–100) at five years.

The evaluation of symptoms varied from 66 ± 4.7 (range 54–72) in the pre-operative period to a score of 86.8 ± 5.6 (range 68–95) at six months, 90.9 ± 3.6 (range 82–95) at 12 months, 92.5 ± 5.1 (range 82–100) at 2 years and 92.1 ± 1.4 (range 85–100) at five years.

The evaluation of the activities of daily life went from 68.1 ± 1.4 (range 56–77) to 90.9 ± 2.8 (range 78–100) to six months to 94.7 ± 2.8 (range 85–100) to 12 months, 95.1 ±2.8 (range 85–100) to 2 years and 94.8 ±4.2 (range 88–100) to five years.

The assessment of the resumption of sporting activities saw a pre-operative score of 43.6 ±7.1 (range 28–55), which rose to 68.5 ± 4.9 (range 45–85) at six months, 72.9 ± 8.5 (range 60–93) at 12 months, 74,4 ± 8.8 (range 60–90) at two years and 75.2 ± 7.9 (range 65–93) at five years.

Finally, the global assessment of the quality of life equal to 38.7 points ± 3.5 (range 30–53) in the pre-operative period and improved markedly already at six months 76.3 points ± 12 (range 63–100) to then reach 77.9 points ± 14.1 (range 64–95) at 12 months, 82.4 points ± 3.5 (range 60–100) at 24 months and 82.1 ± 3.5 (range 65–100) at five years.

**Fig. 4:**
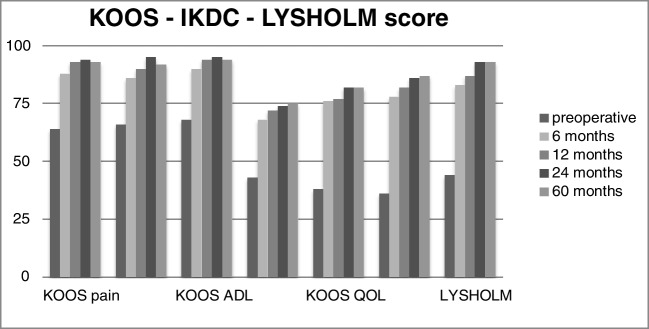
IKDC, Lysholm and KOOS score graphics (pre-op, 6 months, 12 months, 24 months and 60 months). Legend: IKDC-, International Knee Documentation Committee; KOOS-, Knee Injury and Osteoarthritis Outcome Score; QOL-, Quality of Life ADL-, activities of daily living

The VAS score for pain assessment went from a pre-operative value of 6.5 ± 0.7 (range 5–8) to a six month value of 1.1 ± 0.7 (range 0–3) and then further progressed in improvement at 12 months 0.8 ± 0.7 (range 0–3), at two and five years 0.7 ± 0.7 (range 0–3) and five years 0.8 ± 0.8 (range 0–2), respectively (Table 3; Fig. 4).

### Radiological evaluation

**Fig. 5:**
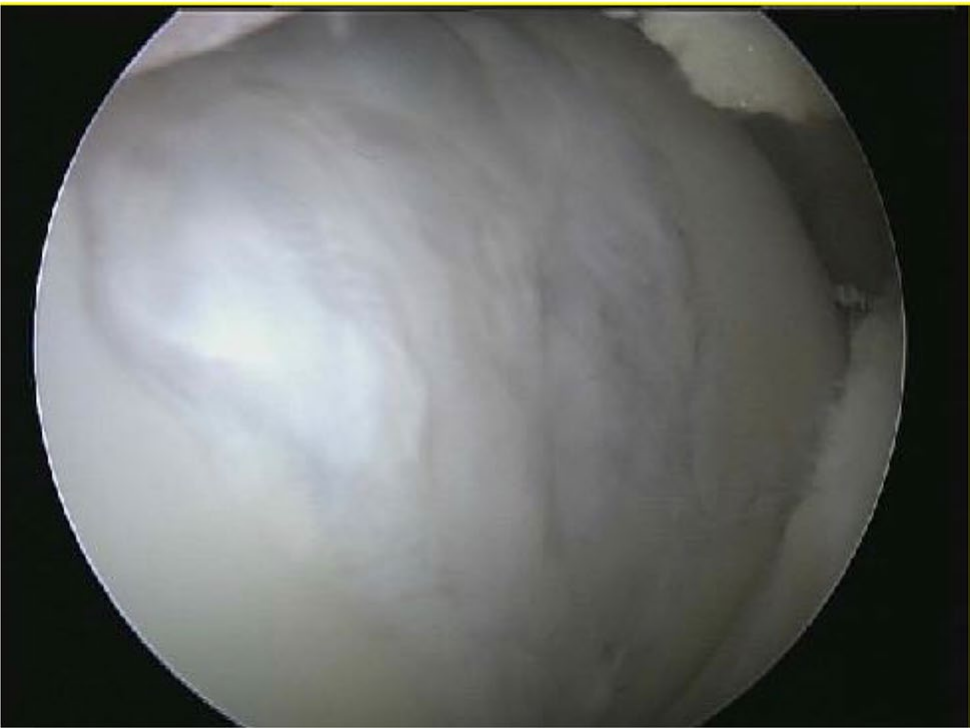
Second-look follow-up image of the trochlear defect treated by LIPO-AMIC procedure showing complete regeneration and filling of the defect

The evaluation of the MR images showed, progressively in the follow-up controls, the neoformation of the subchondral lamina and a significant reduction of the area of the chondral defect, with complete filling of the defect in the majority of cases, in the absence of signs of bone hypertrophy or oedema (Fig. 5).

The mean total MOCART score showed a significative improvement from two year follow-up (69.1 points) to last follow-up that was 81.9 points (range, 30–100 points, SD 24) (Table 4).

**Table 4:** MOCART score evaluation at 24- and 60-month follow-ups

MOCART Score	Follow-up		*P* value
	24 months	60 months	24/60 months
*Mean value, SD (range)*	69.1 ±22.8 (25–100)	81.9 ±24 (30–100)	*p* < 0.05

Complete filling of the defect at the level of the surrounding cartilage was found in 77.8%.

Normal signal intensity of the repair tissue compared with the adjacent native cartilage was seen in 66.7%. A complete integration of the graft was observed in 72.2%. The subchondral lamina was restored in 77.8% of the cases, and intact subchondral bone was observed in 66.7%.

## Discussion

The main finding of this study is that the LIPO-AMIC technique enabled statistically significant and stable outcomes for the treatment of full thickness knee cartilage defects better than what reported in the literature for the AMIC standard technique, by using mesenchymal cells from adipose tissue graft attached to a type I and III double-layer collagen membrane. The positive effects of LIPO-AMIC procedure were observed very early already at the six month postoperative visit, with clinically statistically consistent improvement in pain and functional outcomes.

Focusing on one-step cartilage repair procedures, the literature recently presented long-term reviews of AMIC and OATS procedures [9–11]. In the first [9], the authors reported a Lysholm score improvement by 29 points at two years maintained at last follow-up at nine years, while VAS improved by 3.8 points at two years and 3.9 points at nine years.

Gille et al. [10], in their up to seven year follow-up of AMIC, reported that significant improvement was observed after one and two years (Lysholm + 36.9 points at 1 year, + 37.2 points at 2 years and + 38.1 at 5-year follow-up; VAS 3.2 points improvement at 1 year similarly maintained till 5 years).

The OATS comprehensive review on a total of 610 patients [11] demonstrated from preoperative to final follow-up, Lysholm score mean improvement by 21.1 with a maximum of 30 points increase.

In our series, Lysholm score showed an early significative improvement already at six month follow-up by 38.8, becoming 43.3 at one year and 49.1 at two and five years, while VAS score similarly showed dramatic improvement of 5.4 points at six months and 5.7 points at 12 and 60 months.

Thus, the LIPO-AMIC patients reported greater Lysholm and VAS, starting earlier at six months, but all patients treated by LIPO-AMIC, AMIC and OATS procedures demonstrated to maintain functional and pain improvements both in the medium- and long-term follow-ups.

As stated before, orthobiologic emerging options have become of interest in the attempt to improve the long-term results offered in the treatment of articular cartilage lesions and slow down the progression to OA joint degeneration.

Various studies [12–14] have demonstrated that a collagen scaffold is able to retain mesenchymal cells deriving from microfractures, promote chondrogenesis and consent to obtain a thick and stable repair tissue. In particular, the resorbable Chondro-Gide® membrane, made of type I and III collagen, has the particularity of having a double-layer structure that favours the settlement of cells and their differentiation into the chondrocyte phenotype with production of type II collagen and GAG.

For our purposes, the main interest in adipose tissue mesenchymal cells (ADSCs) stems from the fact that these cells are multipotent, therefore capable of differentiating into different cell types including chondrocytes [15–18]. Even if mesenchymal stem cells isolated from the bone marrow (BMSCs) have similar characteristics to ADSCs, the latter present various advantages, including larger numbers that can be harvested from adipose tissue, easy mini-invasive and less painful withdrawal and less proliferative senescence.

The chondrogenic potential of human ADSCs cells has been clearly demonstrated by various works [19–22]. ADSCs have, therefore, definitely been considered capable of forming cartilage tissue, with the literature confirming the good results obtained with ADSCs both as intra-articular injections [23–26] in the hip and the knee as OA treatment and as orthobiologic complex scaffold reconstruction surgeries [27].

Over the years, to obtain ADSCs, various methods of lipoaspirate processing have been proposed, which can be divided into two broad lines for convenience: the enzymatic and non-enzymatic processing technique [17, 28–30]. The former use enzymes such as collagenase, trypsin or dispase to digest adipose tissue, with a certain variability as regards the number of washes, the concentration of enzymes, the centrifugation and filtration parameters and the erythrocyte lysis methods. ADSC enzymatic harvesting digestion method has represented for long-time the “gold standard”. The enzymatically digested lipoaspirate contains a heterogeneous population of many cell types (preadipocytes, fibroblasts, vascular smooth muscle cells, endothelial cells, resident monocytes/macrophages, lymphocytes, and ADSCs), known as stromal vascular fraction (SVF).

The fraction capable of adhering to the plastic, containing the ADSC, is obtained after the steps and can be further made homogeneous if grown by expansion.

Due to enzyme high costs, side effects and important regulatory and legal concerns on which basis, according to the regulations set by the US Food and Drug Administration (FDA), the fat graft must be minimally manipulated, enzyme-free and used in the same surgical procedure; in the last years, non-enzymatic devices for cell separation based on gentle minimal manipulation and mechanical digestion, centrifugation, separation, fragmentation and concentration have taken the lead [31–38].

These include the Lipogems system, an innovative closed-cycle adipose tissue processing system that allows minimal manipulation of the tissue (only through mechanical action), as permitted by law. The Lipogems® completely closed device permits the fat to be washed, emulsified and micro-fragmented to remove blood and oil residues [39, 40]. The system reduces the adipose cluster dimensions [39, 41–46] that maintain an intact stromal vascular niche, which is considered the cell morphofunctional unit controlling ADSC differentiation [47] and provides ready-to-use micro-fragmented adipose tissue graft and to allow injection with needles of appropriate calibre and the removal of impurities allowing a regenerative product to be immediately available in the room to be applied on the defect and on the scaffold, acting as a slow-released “natural scaffold”, continuously delivering growth factors, extracellular vesicles and much other modulatory information into the microenvironment surrounding damaged cartilage [48].

This work confirmed that the repair technique defined as LIPO-AMIC is able to significantly improve the symptoms and function of the knees affected by full-thickness cartilage defects, starting very early from the first follow-up six months after surgery, thus furtherly validating the use of biologic augmentations for the treatment of focal symptomatic cartilage defects. From the analysis of the data obtained, we also observed a further improvement in symptoms in the subsequent follow-ups up to two years after surgical treatment and a substantial maintenance or slight improvement of the state of well-being after five years.

This study, however, has several limitations. First of all, the authors acknowledge the lack of a control group of patients treated by the standard AMIC technique or with MF, the limited number of patients (18) that although represents the precise desire to follow-up in the following years the initial patients group object of the pilot study conducted in 2017 by the same authors and by itself represents although nearly one third of the total number of patients reported from the data of the AMIC registry (57 patients). Other limitation is represented by the fact that in some cases, the operation was not performed in isolation, but associated with other surgical procedures, as correction of knee alignment or instability, although the final results were similar. Despite the acknowledged limitations, this study highlighted the safety and the potential of the collagenic scaffold and of the autologous adipose tissue derived graft and mesenchymal cells and of their simultaneous use as a combined one-step orthobiologic procedure and, most importantly for the first time, showed good and stable results over time until the last follow-up at five years. Thus, this procedure can be considered as a routine option for the treatment of cartilage defects able to provide durable articular cartilage surface regeneration, with the advantages of easiness and reproducibility of a one-step procedure, reasonable costs and simplified technique respect other two-stage cell-based procedures using autologous chondrocytes.

In this highly specialised surgical procedures, all components are important and their optimal interaction we retain is the key of the success of this procedure.

In fact, the LIPO-AMIC technique improved results in respect to the standard AMIC take advantage of the perfect interaction between the three-dimensional collagen scaffold with microstructural properties capable of favouring direct attachment, proliferation and chondrogenic differentiation of ADSCs which, conversely, have been proved to better differentiate and express their chondrogenic potential when seeded in this type of material, thus exploiting orthobiologics use to improve one-step cartilage repair procedures, by augmented proliferation of cells and extracellular matrix deposition, towards the pathway of cartilage regeneration [49–53].

The enormous work, produced during the last two decades in the laboratories [49–56], represents the positive background of biological augmentation procedures developed in the last years and in particular of the LIPO-AMIC technique.

## Conclusions

Overall, the possibility of obtaining a large number of viable mesenchymal cells from adipose tissue, with intact clusters and stromal vascular niches, with high chondrogenic and paracrine capacity together with the use of a collagen membrane capable of retaining the cells in situ and favouring their chondrogenic transformation, account for the significant improvements found in the patients treated in this study. The functional outcomes evaluation permitted to notice a significant greater and earlier improvement in pain relief, starting from the six month follow-up control and remarkably better improvements than the data reported in the AMIC Registry [5]. In particular, it has to be emphasised that the data obtained by the KOOS score broken down into subgroups showed, together with the confirmation of early, constantly progressive improvement and durature relief of symptoms, the important regain and maintenance of all functional daily life and sport activities at two and especially at five year follow-ups, extraordinarily confirmed by the global assessment of the quality of life raised from 38.7 points in the pre-operative period to 82.4 points at 24 months and remains unchanged at 82.1 at five years.

These results allow to validate the repair of articular cartilage lesions using a resorbable collagen membrane with the concurrent use of adipose tissue graft containing viable autologous adipose tissue–derived stem cells, defined as LIPO-AMIC technique, as a simple, safe and effective one-step procedure able to provide good clinical results and durable cartilage repairs at the medium-term follow-up, even better than what was reported with the standard AMIC technique [5].

However, it is desirable to carry out a clinical and diagnostic imaging study on a larger number of patients with follow-up of longer duration in order to be able to further confirm and validate the potential of this reconstructive and regenerative surgical procedure even in the long-term follow-up.
